# Prolonging hormone sensitivity in prostate cancer xenografts through dual inhibition of AR and mTOR

**DOI:** 10.1038/sj.bjc.6605882

**Published:** 2010-09-14

**Authors:** A Schayowitz, G Sabnis, O Goloubeva, V C O Njar, A M H Brodie

**Affiliations:** 1 Department of Pharmacology and Experimental Therapeutics, School of Medicine, University of Maryland, Health Science Facility I, Room 580G, 685 West Baltimore Street, Baltimore, MD 21201, USA; 2 Department of Epidemiology and Biostatistics, Baltimore, MD 21201, USA; 3 University of Maryland Marlene and Stewart Greenebaum Cancer Center, Baltimore, MD 21201, USA

**Keywords:** androgen receptor, cross-talk, mTOR, anti-androgens, signal transduction inhibitors

## Abstract

**Background:**

To determine the mechanisms associated with loss of androgen dependency and disease progression in prostate cancer (PCa), we investigated the relationship between the androgen receptor (AR) and mTOR pathways and the impact of inhibiting both pathways in androgen-dependent and castration-resistant PCa models.

**Experimental design:**

Androgen-dependent (LNCaP) and castration-resistant PCa (HP-LNCaP) cells were grown as tumours in SCID mice. Once tumours reached 500 mm^3^, animals were grouped and injected subcutaneous with vehicle, our novel anti-androgen/androgen synthesis inhibitor, VN/124-1, bicalutamide, and everolimus. Tumour volumes were measured biweekly. The PSA and protein analyses were performed after completion of the treatment.

**Results:**

The addition of everolimus to bicalutamide treatment of resistant tumours significantly reduced tumour growth rates and tumour volumes. Anti-androgen treatment also increased protein expression of multiple signal transduction pathways earlier than vehicle-treated control xenografts. VN/124-1 plus everolimus acted in concert to reduce tumour growth rates in our castration-resistant xenograft model.

**Conclusions:**

This study suggests that dual inhibition of AR and mTOR in castration-resistant xenograft models can restore sensitivity of tumours to anti-androgen therapy. Furthermore, after bicalutamide failure, dual inhibition with VN/124-1 and everolimus was the most effective treatment.

The progression to castration-resistant prostrate cancer (CRPC) requires the ability to overcome two major roadblocks, the initial survival of prostate cancer (PCa) cells during the acute onset of androgen deprivation and the ability to proliferate in a castrate-independent manner. During castrate-resistant progression, PCa cells adapt to alternative cellular signalling to survive in an androgen-depleted environment.

The lack of successful treatments for CRPC is, in part, a reflection of the poor understanding of the mechanisms and pathways that lead to increased cell survival after androgen ablation. Several mechanisms and pathways have been proposed to explain castrate resistance. The two main mechanisms of progression are those that involve the androgen receptor (AR) and those that bypass the AR. However, these pathways are not mutually exclusive, and cross-talk between the AR and signal transduction pathways (STPs) in CRPC has been demonstrated (Baron *et al*, 2004; Cinar *et al*, 2005; Culig *et al*, 2005; Ghosh *et al*, 2005; Edwards and Bartlett, 2005a, 2005b).

We and others have shown that PCa cells can become hypersensitive to androgens as a means of circumventing androgen blockade (Gregory *et al*, 2001a, 2001b). Hypersensitivity is believed to be caused by an increase in AR expression, which is a result of AR gene amplification (Visakorpi *et al*, 1995; Linja *et al*, 2001; Edwards *et al*, 2003; Chen *et al*, 2004). Koivisto *et al* (1997) originally showed that AR amplification contributed directly to the failure of androgen deprivation by allowing cells to grow in low concentrations of androgen, resulting in hypersensitivity (Gregory *et al*, 2001a, 2001b). Numerous studies have shown that despite the elimination of testicular androgens, the level of T and DHT in recurrent PCa tissue is unchanged relative to levels in benign tissue (Mohler *et al*, 2004; Titus *et al*, 2005). Intraprostatic androgen concentrations are therefore likely to be more precise indicators of the tumour environment than serum hormone levels (Habib *et al*, 1976; Heracek *et al*, 2007). Furthermore, it has been shown that DHT levels in prostatic tissue of castrated patients are sufficient to activate the AR and stimulate expression of androgen-regulated genes (Culig *et al*, 1999; Mohler *et al*, 2004; Mostaghel *et al*, 2007).

The exact mechanisms of steroids in promoting proliferation in CRPC remain unclear. However, there is increasing evidence to suggest that compensatory signalling mechanisms and cross-talk occur between growth factor receptor pathways and AR in androgen-dependent and CRPC cell lines. Recent investigations have elucidated multiple mechanisms for regulation of AR by STPs and *vice versa* (Cinar *et al*, 2005; Yang *et al*, 2005). Several mechanisms of cross-talk have been reported between the AR and P13K/Akt/mTOR signalling pathway. Indeed, we have previously reported that the P13K/Akt/mTOR pathway is upregulated in LNCaP and HP-LNCaP cells (Schayowitz *et al*, 2008). Although the specific therapeutic impact is unknown, synergy between AR antagonists and P13K/Akt/mTOR inhibitors further suggests a functional relationship between the two pathways (Lin *et al*, 1999, 2001, 2004; Manin *et al*, 2002; Sun *et al*, 2003; Festuccia *et al*, 2005; Yang *et al*, 2005; Schayowitz *et al*, 2008).

From our previous *in vitro* findings (Schayowitz *et al*, 2008), we concluded that growth factor receptor pathways, specifically P13K/Akt/mTOR, are upregulated as LNCaP androgen-dependent cells adapt to become castrate resistant. We found that blocking both pathways with AR plus mTOR inhibitors was more effective that either inhibitor alone in reducing proliferation (Schayowitz *et al*, 2008). In this report, we have investigated whether tumour growth of apparent castrate-resistant tumours could be effectively inhibited by combining an mTOR inhibitor (everolimus, RAD-001, a rapamycin analogue inhibitor that blocks the downstream signalling cascade *in vitro* and *in vivo* (Boulay *et al*, 2004; Vignot *et al*, 2005)) with our novel compound VN/124-1. VN/124-1 is a potent inhibitor of CYP17 and reduces androgen synthesis. It also binds to the AR and causes its degradation. VN/124-1 is at least as effective as current anti-androgens in androgen-dependent PCa cell lines and significantly more effective in CRPC cells. Combined with inhibitors of STPs, this compound may be an effective strategy for treatment of CRPC (Schayowitz *et al*, 2008).

## Materials and methods

### Materials

RPMI, T-medium, penicillin/streptomycin solution (10 000 IU each), 0.25% trypsin-EDTA (1 mmol l^−1^) solution, and Dulbecco's phosphate-buffered saline were obtained from Invitrogen (Carlsbad, CA, USA). Regular serum and charcoal-stripped fetal bovine serum (FBS) were obtained from Hyclone (Logan, UT, USA). The C4-2B cells were kindly supplied by Dr Yun Qiu (University of Maryland, Baltimore, MD, USA). Dimethyl sulfoxide (DMSO), 3-(4,5-dimethylthiazol-2-yl)-2,5-diphenyltetrazolium bromide (MTT), and Tween 20 were obtained from Sigma Chemicals Company (St. Louis, MO, USA). The ECL chemiluminescence kit and Hybond-ECL nitrocellulose membranes were purchased from Amersham Biosciences (Piscataway, NJ, USA). Antibodies against p-Akt (Ser-473 and Thr-308), p-mTOR (Ser2448 and Ser2481), p-MAPK, IGFR1, p-P70S6K, p-PS6, immobilised Akt, and β-actin were purchased from Cell Signaling Technology, Beverly, MA, USA. The p-HER2 was purchased from Upstate (Temecula, CA, USA) and AR (SC-441) was obtained from Santa Cruz Biotechnology (Santa Cruz, CA, USA). The total PSA ELISA kit was purchased from Diagnostic Systems Laboratories in Webster (TX, USA). Bicalutamide (Casodex) was kindly provided by Dr Elizabeth Anderson (Astra Zeneca, Macclesfield, UK). Everolimus (42-*O*-(2-hydroxy) ethyl-rapamycin) (RAD-001) was kindly provided by Dr D Evans (Novartis Pharmaceuticals, Basel, Switzerland), and dissolved in 100% DMSO for cell culture application. Novel compound VN/124-1 (3*β*-hydroxy-17-(1*H*-benzimidazol-1-yl)androsta-5,16-diene) was designed and synthesised by one of us, Dr Vincent CO Njar (Handratta *et al*, 2005).

### Cell culture

Androgen-dependent PCa cells derived from metastatic lymph node of a PCa patient (LNCaP) were routinely maintained in RPMI 1640 medium with 10% FBS and 1% penicillin/streptomycin solution (Horoszewicz *et al*, 1983). The CRPC (HP-LNCaP) cells were created through regular passaging of the LNCaP cell line for more than 1 year, as previously described and characterised (Schayowitz *et al*, 2008). Low-passage LNCaP cells (LNCaP) were designated as under passage 25, whereas high-passage cells (HP-LNCaP) were over passage 75. Both lines were maintained in identical conditions. The CRPC *in vivo*-derived cell line from LNCaP cells (C4-2B) were maintained in T-Medium with 10% FBS and 1% penicillin/streptomycin (Thalmann *et al*, 1994, 2000).

### Cell growth studies

MTT proliferation assays were performed to examine the effect of R1881, a synthetic, non-metabolised androgen, on cell proliferation in LNCaP and HP-LNCaP cell lines. The IC_50_ values for inhibition were calculated via non-linear regression. All growth studies involving anti-androgen were performed in the presence of 0.1 nM R1881.

### Western blotting

The protein extracts from tumours were prepared by pelleting homogenised tissue suspended in ice-cold DPBS and lysed with cell lysis buffer containing protease inhibitors, Tris-CL, and Triton X-100. Equal amounts (50 *μ*g) of protein from each sample were separated on a denaturing polyacrylamide gel, transferred to nitrocellulose membrane, and western blotting was carried as previously described (Schayowitz *et al*, 2008). The densitometric values are corrected for loading control shown in the bottom panel of the figures. All western blots were performed at least two times; blots shown are representative of all results.

### PSA ELISA

Tissue lysates and serum were loaded in the total PSA ELISA Kit (DSLabs, Webster, TX, USA) in triplicates to determine PSA concentrations. The assay standards, controls, and samples were incubated in 96-well plates following the manufacturer's instructions. Each original experimental sample was assayed in triplicate. The PSA values (ng ml^−1^) were determined using log–log curve fit to the standard curve.

### Tumour growth in male SCID mice

All animal studies were performed according to the guidelines and approval of the Animal Care Committee of the University of Maryland School of Medicine. Male SCID mice 4–6 weeks of age were obtained from the National Cancer Institute-Frederick Cancer Research Center (Frederick, MD, USA). The mice were housed in a pathogen-free environment under controlled conditions of light and humidity and received food and water *ad libitum*. The mice were inoculated with LNCaP or HP-LNCaP cells. These *in vivo* studies were performed using two PCa cell lines. Androgen-dependent cells, designated as LNCaP cells, and androgen-independent cells, denoted as HP-LNCaP cells (Schayowitz *et al*, 2008), were inoculated into male SCID mice and formed tumours. The mice were then treated with a combination of inhibitors to block the AR and mTOR activation. The cells were grown in regular growth conditions (RPMI, 10% FBS and 1% penicillin/streptomycin). Subconfluent cells were scrapped into DPBS, collected by centrifugation, and resuspended in Matrigel (10 mg ml^−1^) at 2.0 × 10^7^ cells per ml. Each mouse received a subcutaneous (s.c.) inoculation at one site per flank with 100 *μ*l of cell suspension. Tumour formation varied between 4 and 8 weeks by cell line, although on tumours reaching 500 mm^3^, mice were grouped by 5 or 10 with equal tumour volumes. Tumour size was measured twice weekly with calipers, and tumour volume was calculated by the formula 4/3*πr*_1_^2^ × *r*_2_, where *r*_1_ is the smaller radius. Treatments varied between 3 and 7 weeks by experiment. All mice were killed by decapitation under anaesthesia, and the trunk blood was collected for PSA analysis. Tumours were also excised, cleaned, weighed, and stored in −80°C for additional analysis.

### Statistics

Data on tumour volume and weight were analysed separately. Growth curve models were used to summarise the pattern of response over treatment duration. The tumour growth rate (the slope of regression line) was compared between pre-specified treatment groups. The mean tumour volume was modelled as a combination of fixed effects shared by all subjects and random effects unique to a particular animal. We used random effects for both intercept and slope at the animal level. The proportions of tumours that shrunk below a threshold during treatment were compared using Fisher's exact test. The treatment groups were compared with one another at 0.05 level of statistical significance. All reported *P*-values were two sided. Mixed-effects models were used to compare average tumour volume on pre-specified time points. The Tukey–Kramer method was used to adjust for multiple comparisons.

## Results

### *In vitro* effects of androgens on LNCaP and HP-LNCaP

**Figure 1:**
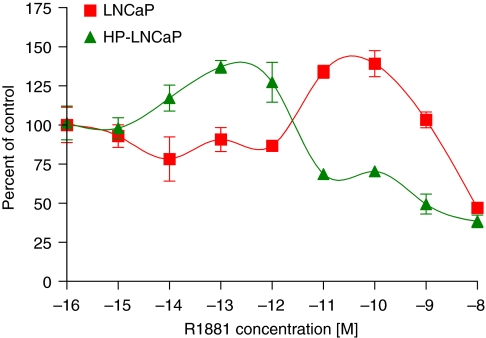
The effect of androgen on LNCaP and HP-LNCaP cell lines. Cell lines were treated with increasing concentrations of R1881 and assayed on day 8 using the MTT assay as described in Materials and Methods. Cell stimulation is expressed as percent control compared with vehicle-treated control (mean±s.e.).

As we previously reported, the proliferation of HP-LNCaP cells was significantly greater than that of LNCaP cells in the presence and absence of steroids (Schayowitz *et al*, 2008). The LNCaP cells demonstrated a biphasic response to the synthetic androgen, R1881 (Figure 1). Growth was stimulated by ∼50% at 10^−10^ M in LP-LNCaP cells and inhibited by 50% at 10^−9^ M in HP-LNCaP cells. The HP-LNCaP cell proliferation was stimulated in a biphasic manner, although the dose response curves were shifted 3-log to the left (Figure 1). Thus, HP-LNCaP cells were stimulated by 50% at 10^−13^ M. Proliferation was inhibited at concentrations above 10^−10^ M. These results suggests that HP-LNCaP cells are hypersensitive to androgens and can respond to very low levels of androgens, thus contributing to their apparent androgen independence. These conclusions are supported by our previous observation that AR levels were increased in HP-LNCaP cells compared with LNCaP cells (Schayowitz *et al*, 2008). However, the absolute values differ between the different assay systems used in the different studies.

### The effects of an anti-androgens and mTOR inhibition on growth of LNCaP tumours

The effect of treatment with AR and mTOR inhibitors was compared with the addition of an mTOR inhibitor to bicalutamide therapy, as tumours progressed on the anti-androgens. A total of 40 male SCID mice were inoculated with LNCaP cells and monitored weekly for tumour formation. When tumours reached 500 mm^3^, mice were then grouped so that tumour volumes were not significantly different (*P*=0.66) and treated with bicalutamide (100 mg kg^−1^ q.d. s.c.) or everolimus (2.5 mg kg^−1^ every 2 weeks p.o.). When tumours had doubled in volume (tumour progression) on day 7, animals in the bicalutamide group were subgrouped (*n*=10) by equal tumour volume and continued on anti-androgen only or administered everolimus (2.5 mg kg^−1^ every 2 weeks p.o.) in addition to bicalutamide (100 mg kg^−1^ q.d. s.c.).

**Figure 2:**
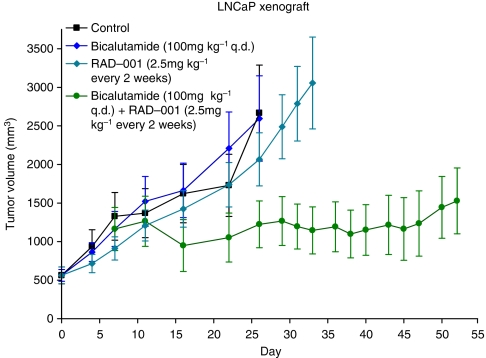
The effect of bicalutamide, everolimus, and bicalutamide plus everolimus on the growth of LNCaP xenografts. Each mouse received s.c. inoculation at one site in each flank with 100 *μ*l of LNCaP cells suspended in Matrigel. On tumour formation, mice were grouped and injected daily with vehicle, bicalutamide (*n*=20) (100 mg kg^−1^ q.d. s.c.) or everolimus (*n*=10) (2.5 mg kg^−1^ every 2 weeks p.o.). Tumour measurements began at ∼500 mm^3^ and tumour volumes were measured biweekly. After disease progression (tumour volume doubling), the bicalutamide (*n*=20) group was split into bicalutamide (*n*=10) (100 mg kg^−1^ q.d. s.c.) and bicalutamide plus everolimus (*n*=10) (100 mg kg^−1^ q.d. s.c. + 2.5 mg kg^−1^ every 2 weeks p.o.). Everolimus plus bicalutamide was significantly different (*P*<0.04) from all other treatments and control.

As seen in Figure 2, tumour growth was controlled in the group administered bicalutamide plus everolimus, whereas tumours continued to grow during treatment with bicalutamide and everolimus alone. Inhibition of tumour growth in the combination treatment group was maintained for over 50 days.

Animals in the control and bicalutamide group were monitored for over 26 days, the everolimus group for over 33 days, and mice treated with bicalutamide plus everolimus were studied for 65 days. Linear mixed-effects models were used to analyse the data. Random effects for intercept and slope at the tumour level nested within a mouse were used to fit the model. Data on tumour volume were log transformed to satisfy the assumptions of the linear mixed-effects models. There were no differences in average tumour volume on day 0 across treatment groups; *P*=0.66. The average tumour volume on day 7 was not different between mice that stayed on bicalutamide and the group administered everolimus in addition to bicalutamide; *P*=0.59. By day 26, the growth rates between bicalutamide plus everolimus and control (*P*<0.0001) or bicalutamide (*P*<0.0001) were significantly reduced. Animals treated with everolimus alone, compared with everolimus added to bicalutamide, also had statistically different growth rates (*P*=0.04).

The results suggest that in the LNCaP xenograft model, the addition of everolimus to bicalutamide treatment of resistant tumours was more effective than either drug alone, and restored the sensitivity of tumours to anti-androgen therapy.

### The effects of anti-androgens on the expression of proteins in STPs in HP-LNCaP tumours

**Figure 3:**
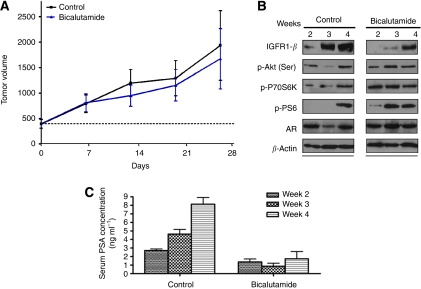
(**A**) The effect of bicalutamide on the growth of HP-LNCaP xenografts. Each mouse received s.c. inoculation at one site in each flank with 100 *μ*l of HP-LNCaP cells suspended in Matrigel. On tumour formation, mice were grouped and injected daily with vehicle (*n*=15) or bicalutamide (*n*=15) (100 mg kg^−1^ q.d. s.c.). Measurements began at ∼400 mm^3^ and tumour volumes were measured weekly. Five mice from each group were isolated and killed at weeks 2, 3, and 4 for tumour and PSA analysis. Tumour growth rates were not significantly different between bicalutamide treatment and control groups. (**B**) Expression of signalling proteins in tumour lysates from HP-LNCaP xenografts. Mice described above in **A** were treated with vehicle (*n*=10) and bicalutamide (*n*=10). Tumours were collected at weeks 2, 3, and 4 of treatment. Western immunoblotting analysis is described in Materials and Methods. (**C**) Serum PSA levels of HP-LNCaP xenografts. Total PSA ELISA from the serum of HP-LNCaP xenografts treated with vehicle or bicalutamide for 2, 3, or 4 weeks. The experimental protocol is described in Materials and Methods. The PSA values for bicalutamide-treated mice were statistically significantly different from controls at all weeks of treatment (*P*<0.005).

We sought to determine the molecular impact of the anti-androgen treatment over time in a CRPC model. For this investigation, we analysed tumours collected at the same time points. A total of 30 male SCID mice were inoculated with HP-LNCaP cells. Measurable tumours formed in ∼4 weeks after inoculation. Mice were then grouped (*n*=15) and were treated with vehicle (control) or bicalutamide (100 mg kg^−1^ q.d. s.c.), and tumours were measured weekly (Figure 3A). Mice (*n*=5) with equal tumour volumes were isolated at three different time points (2, 3, and 4 weeks) and killed. Tumours were homogenised and prepared for protein analysis as described in Materials and Methods.

Tumours of mice treated with anti-androgens were minimally responsive as expected, as these mice were inoculated with androgen-resistant cells (Figure 3A). There was an increase in expression of proteins in the signalling pathways over time in control and treated groups. By 4–6 weeks, all proteins measured in the signalling pathways were increased. Additionally, bicalutamide treatment induced increased expression of p-Akt, p-P70S6K, and p-PS6 faster than in control mice. Changes in expression of p-HER2, p-MAPK, and p-mTOR at 4 weeks (data not shown) were about the same in control and bicalutamide-treated tumours. Serum was also collected for PSA analysis. As in tissue, serum PSA levels increased after 4 and 6 weeks compared with levels at 2 weeks in control mice. However, PSA levels in the serum, but not tissue levels, were greatly reduced at all time periods in the bicalutamide-treated group compared with controls (Figure 3C). These results suggest that serum PSA level did not reflect PSA levels in the tumour tissue or the growth of tumour.

### The effect of anti-androgens and mTOR inhibitor on HP-LNCaP xenografts

The rationale for this *in vivo* investigation was based on our previously described *in vitro* findings, which suggested that, unlike bicalutamide, VN/124-1 is effective in hormone-dependent and CRPC models (Schayowitz *et al*, 2008). VN124-1 may also be more effective in blocking tumour growth in the CRPC model, in combination with mTOR inhibition (Schayowitz *et al*, 2008).

A total of 30 male SCID mice were inoculated with HP-LNCaP cells. Approximately 4 weeks later, on tumour formation, mice were divided into six groups (*n*=5) and treatment was initiated. The six groups included control, VN/124-1, bicalutamide, everolimus, bicalutamide plus everolimus, and VN/124-1 plus everolimus.

**Figure 4:**
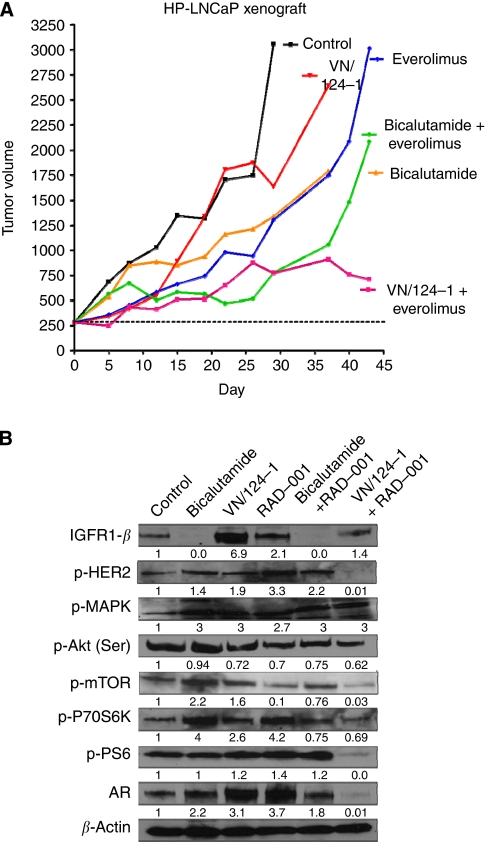
(**A**) The effect of bicalutamide, VN/124-1, everolimus, bicalutamide plus everolimus, and VN/124-1 plus everolimus on the growth of HP-LNCaP xenografts. Each mouse received s.c. inoculation at one site in each flank with 100 *μ*l of LNCaP cells suspended in Matrigel. On tumour formation, mice were grouped and injected daily with vehicle (*n*=5), bicalutamide (100 mg kg^−1^ q.d. s.c.; *n*=5), VN/124-1 (50 mg kg^−1^ b.i.d. s.c.; *n*=5), everolimus (2.5 mg kg^−1^ every 2 weeks p.o.; *n*=5), bicalutamide (100 mg kg^−1^ q.d. s.c.) + everolimus (2.5 mg kg^−1^ every 2 weeks p.o.) (*n*=5) or VN/124-1 (50 mg kg^−1^ b.i.d. s.c.) + everolimus (2.5 mg kg^−1^ every 2 weeks p.o.) (*n*=5). Measurements began at ∼250 mm^3^ and tumour volumes were measured biweekly. For clarity, the s.e. bars have been omitted, but full statistical analysis was performed on tumour measurements. Pairwise comparisons on days 29 and 43 were significantly different (*P*<0.03) between everolimus and VN/124-1 plus everolimus. There was no significant difference between everolimus plus bicalutamide and everolimus plus VN/124-1. (**B**) Expression of signalling proteins from HP-LNCaP xenografts. Tumours were collected at autopsy from mice described above in **A**. Animals were treated with vehicle (*n*=5), bicalutamide (*n*=5), VN/124-1 (*n*=5), everolimus (*n*=5), bicalutamide plus everolimus (*n*=5), or VN/124-1 plus everolimus (*n*=5). Western immunoblotting analysis of tumour lysates is described in Materials and Methods. Representative blots are shown.

Control tumours grew rapidly and mice were killed 27 days after the start of the treatment (Figure 4A). VN/124-1 (50 mg kg^−1^ b.i.d. s.c.) was effective for 15 days, demonstrating a slower trend in tumour growth compared with control. However, after day 15, tumours had increased growth rates and mice were killed at day 36. Everolimus (2.5 mg kg^−1^ every 2 weeks p.o.) was effective in reducing tumour volume for the first 30 days of the experiment, after that tumour growth was rapid and exponential. Dual inhibition of bicalutamide plus everolimus was more effective than either bicalutamide or everolimus alone at day 35 (Figure 4A). However, comparable with all other treatment groups, once treatment failed, tumour growth was rapid. The bicalutamide plus everolimus treatment group had a tumour volume over 300% at day 41 (Figure 4). Inhibition of tumour growth with VN/124-1 plus everolimus was comparable with bicalutamide plus everolimus for the first 36 days of treatment. However, whereas tumours became resistant to the bicalutamide plus everolimus therapy, they remained sensitive to VN/124-1 plus everolimus. Tumour volumes were <750 mm^3^ after 43 days at termination of the experiment (Figure 4A). All treatments were well tolerated and there were no changes in body weight of the treated animals over the duration of the experiment.

For statistical analysis, animals in the control group were monitored over 29 days. In the VN/124-1 and bicalutamide groups, tumours were measured for 37 days. The three groups receiving everolimus administration were studied for 43 days. The tumour growth rate (slope) was compared between the controls and each of the five treatment groups. The growth curves were estimated over 29 days, but there were no differences found in growth rate among these groups. However, pairwise comparisons of mean tumour volume on days 29 and 43 demonstrated a statistically significant difference between VN/124-1 plus everolimus and everolimus (*P*<0.003) and also bicalutamide (*P*<0.0001). Therefore, the addition of VN/124-1 to everolimus therapy proved to be superior to everolimus therapy alone in the HP-LNCaP model. There was no increase in tumour growth after day 25 in the VN/124-1 plus everolimus-treated group (Figure 4A).

Serum and tumour PSA levels were also analysed. As expected, PSA concentrations in serum were significantly lower than in the tumour tissue. Mean PSA in serum was 8.36 ng ml^−1^ and 454.4 ng ml^−1^ in tissue from the control mice. Serum PSA after VN/124-1 treatment was 4.8 ng ml^−1^ compared with bicalutamide and everolimus values of 7.4 ng ml^−1^ and 6.7 ng ml^−1^, respectively. The mean serum PSA levels from the VN/124-1 plus everolimus treatment group were undetectable. However, the mean PSA level in tissue (214.1 ng ml^−1^), in the combination of VN/124-1 plus everolimus group, was also statistically significantly lower than all other treatment groups.

On termination of treatment, mice were killed, tumours were removed, and lysed in preparation for western immunoblotting. Compared with control mice, treatment with bicalutamide increased expression of p-HER2 (1.4-fold), p-MAPK (3-fold), p-mTOR (2.2-fold), and p-P70S6K (4-fold) (Figure 4B). Interestingly, bicalutamide reduced IGF-1R expression significantly, compared with control and VN/124-1. VN/124-1 tumours demonstrated increase in growth factor signalling with an increase in expression of IGFR1 (6.9-fold), p-HER2 (1.9-fold), p-MAPK (3-fold), p-mTOR (1.6-fold), p-P70S6K (2.6-fold), and p-PS6 (1.2-fold) (Figure 4B). The AR protein expression was increased in these groups, consistent with increased sensitivity to androgens. Everolimus treatment resulted in a 90% decrease in p-mTOR. However, there were increases in both up- and downstream proteins, including IGFR1, p-HER2, p-MAPK, p-P70S6K, and p-PS6. The combination therapy of bicalutamide plus everolimus reduced IGFR1 (100%), p-Akt (25%), p-mTOR (25%), and p-P70S6K (25%), but increased expression of p-HER2 (120%), p-MAPK (300%), p-PS6 (20%), and AR (80%) (Figure 4B). Tumour growth was well controlled by VN/124-1 plus everolimus, and this combination resulted in reduced expression of most proteins. Thus, dual inhibition resulted in a decrease of p-HER2 (100%), p-Akt (40%), p-mTOR (97%), p-P70S6K (40%), and p-PS6 (100%) downstream of mTOR, as well as AR (100%) (Figure 4B). The IGFR1 was slightly increased compared with control, but lower than the high level due to VN/124-1 treatment alone. The p-MAPK was increased by all treatments compared with control. The increase in p-MAPK due to anti-androgen therapy is consistent with *in vitro* findings (Schayowitz *et al*, 2008).

## Discussion

Lack of effective treatment of CRPC represents a significant problem in current management of PCa. Despite initial responses to hormonal therapy, nearly all advanced PCa patients eventually fail treatment, as tumours become resistant. This investigation was designed to understand the molecular mechanisms that enable androgen-dependent PCa cells to progress to CRPC. We also sought to determine whether blocking the resistance pathway would restore sensitivity to standard anti-androgens and/or to our novel agent VN/124-1. Improved understanding of the interaction between the AR and growth factor signalling could yield a better strategy for treating disease progression, which may delay, re-sensitise, or prevent CRPC. The data suggest that increased sensitivity to androgens and a compensatory cross-talk mechanism between the AR and mTOR could have a role in the progression to androgen independence. In the two models used here, resistance to androgen deprivation was developed *in vitro* in HP-LNCaP cells and *in vivo* in the LNCaP tumours with anti-androgen treatment. As shown in Figure 2, LNCaP tumours were resistant to bicalutamide after 7 days. However, when everolimus was added to the anti-androgen therapy at 7 days, tumour growth was effectively inhibited. Thus, the addition of everolimus to bicalutamide treatment of resistant tumours significantly decreased growth rate and tumour volume compared with single-agent bicalutamide (*P*=0.0001) or everolimus (*P*=0.04). Additionally, in the HP-LNCaP xenograft model (Figure 4A), dual inhibition from the beginning with everolimus (5 mg kg^−1^ q.w. p.o.) plus VN/124-1 (100 mg kg^−1^ q.d. s.c.) significantly (*P*=0.03) reduced tumour volumes and growth rates compared with single-agent everolimus. Furthermore, growth trends indicate that VN/124-1 plus everolimus was more effective than bicalutamide plus everolimus. Although PSA levels in tumour tissue were higher than in serum, nevertheless, PSA levels in serum and tissues were reduced only by VN/124-1 plus everolimus, consistent with tumour growth measurements, and indicate the greater anti-tumour efficacy of VN/124-1 than bicalutamide. It is of note that, although serum levels were reduced to undetectable levels, tissue levels were still measurable, consistent with incomplete tumour suppression.

VN/124-1 inhibits androgen synthesis, as well as binds to the AR and causes its downregulation (Vasaitis *et al*, 2008). Inhibition of androgen synthesis, resulting in AR instability in the presence of everolimus, may have contributed to the greater efficacy of the combination of everolimus and VN/124-1 over everolimus and bicalutamide in the intact mice used here. Nevertheless, VN/124-1 alone did not decrease AR levels in the HP-LNCaP tumours. However, the tumours were collected at autopsy and were clearly resistant, very large, and actively growing on treatment with VN/124-1. This is consistent with the AR still being present in the HP-LNCaP-resistant tumours. In tumours treated with VN/124-1 in combination with everolimus, AR was markedly reduced and tumour growth inhibited.

*In vivo* findings also demonstrate that inhibition of AR via bicalutamide or VN/124-1 increases the expression and activation of several growth factor signalling proteins. Compensatory cross-talk was evident by changes in proteins in AR, the MAPK, and P13K/Akt/mTOR pathways, Thus, inhibition of mTOR increased AR protein expression *in vitro* as well as *in vivo* (Schayowitz *et al*, 2008). Furthermore, inhibition of AR resulted in increased expression of IGFR1, p-HER2, and p-mTOR *in vivo*. We found that serum PSA levels, but not tissue levels, were suppressed by bicalutamide and VN/124-1, although tumour growth was not inhibited. This suggests that tissue PSA may reflect tumour growth more accurately, and that only a portion of the PSA produced enters circulation. It is also possible that AR-regulated PSA is still responsive to anti-androgens, whereas tumour growth is independent of androgens and is responsive to other signalling pathways such as mTOR.

Our results indicate that blockade of AR with anti-androgens and of mTOR with everolimus improved sensitivity to these agents. Although others (Kaarbø *et al*, 2010) have found that the PI3K pathway is increased in PCa, there was little effect of the anti-androgens or everolimus on PI3K expression. However, the combination of VN/124-1 and everolimus significantly reduced AR, indicating cross-talk between these signalling pathways. Although the inhibition of AR and signal transduction proteins with bicalutamide plus everolimus is effective, treatment with VN/124-1 in combination with everolimus was superior in maintaining tumour growth suppression in the HP-LNCaP xenograft. This is consistent with previous findings that bicalutamide is less effective than VN/124-1 as single-agent therapy, (Schayowitz *et al*, 2008).

In summary, our studies show that interaction between the AR and mTOR pathways appears to be involved in tumour progression in the xenograft models. There is an increase in signal transduction protein expression as tumours progress on anti-androgen therapy and in control tumours that are actively growing. Bicalutamide and everolimus treatment reduced tumour growth to some extent in comparison with that of control tumours in the resistant models. However, by combining mTOR inhibition with an anti-androgen to block both the pathways from the beginning, tumour growth was statistically significantly reduced compared with the effect of these drugs alone in the LNCaP and HP-LNCaP xenografts. Thus, there may be value in using an mTOR inhibitor in combination with an AR downregulator such as VN/124-1. VN/124-1 in combination with an mTOR inhibitor may reduce AR levels and thereby delay transition to growth factor receptor signalling and hormone resistance. Although everolimus in combination with bicalutamide has not been found to be effective in PCa patients (Buckle *et al*, 2010), our findings suggest that the combination of everolimus with VN/124-1 may be effective in resistant disease, based on the more complete inhibition of AR and mTOR signalling. Improved understanding of the interaction between the AR and alternate signalling pathways could yield a better strategy for treating tumour progression that may delay, re-sensitise, or prevent CRPC.

## References

[bib1] Baron S, Manin M, Beaudoin C, Leotoing L, Communal Y, Veyssiere G, Morel L (2004) Androgen receptor mediates non-genomic activation of phosphatidylinositol 3-OH kinase in androgen-sensitive epithelial cells. J Biol Chem 279: 14579–14586 14668339 10.1074/jbc.M306143200

[bib2] Boulay A, Zumstein-Mecker S, Stephan C, Beuvink I, Zilbermann F, Haller R, Tobler S, Heusser C, O′Reilly T, Stolz B, Marti A, Thomas G, Lane HA (2004) Antitumor efficacy of intermittent treatment schedules with the rapamycin derivative RAD001 correlates with prolonged inactivation of ribosomal protein S6 kinase 1 in peripheral blood mononuclear cells. Cancer Res 64: 252–261 14729632 10.1158/0008-5472.can-3554-2

[bib3] Buckle GC, Werner L, Oh WK, Bubley G, Hayes JH, Weckstein D, Elfiky A, Sims DM, Kantoff P, Taplin M (2010) Phase II trial of RAD001 (R) and bicalutamide (B) for castration-resistant prostrate cancer (CPRC). J Clin Oncol 28(suppl): 7s, abstract 4660 10.1111/j.1464-410X.2012.11456.x 22928480

[bib4] Chen CD, Welsbie DS, Tran C, Baek SH, Chen R, Vessella R, Rosenfeld MG, Sawyers CL (2004) Molecular determinants of resistance to antiandrogen therapy. Nat Med 10: 33–39 14702632 10.1038/nm972

[bib5] Cinar B, De Benedetti A, Freeman MR (2005) Post-transcriptional regulation of the androgen receptor by mammalian target of rapamycin. Cancer Res 65: 2547–2553 15805247 10.1158/0008-5472.CAN-04-3411

[bib6] Culig Z, Hoffmann J, Erdel M, Eder IE, Hobisch A, Hittmair A, Bartsch G, Utermann G, Schneider MR, Parczyk K, Klocker H (1999) Switch from antagonist to agonist of the androgen receptor bicalutamide is associated with prostate tumour progression in a new model system. Br J Cancer 81: 242–251 10496349 10.1038/sj.bjc.6690684 PMC2362859

[bib7] Culig Z, Steiner H, Bartsch G, Hobisch A (2005) Mechanisms of endocrine therapy-responsive and -unresponsive prostate tumours. Endocr Relat Cancer 12: 229–244 15947099 10.1677/erc.1.00775a

[bib8] Edwards J, Bartlett JMS (2005a) The androgen receptor and signal-transduction pathways in hormone-refractory prostate cancer. Part 2: androgen-receptor cofactors and bypass pathways. BJU Int 95: 1327–1335 15892826 10.1111/j.1464-410X.2005.05527.x

[bib9] Edwards J, Bartlett JMS (2005b) The androgen receptor and signal-transduction pathways in hormone-refractory prostate cancer. Part 1: modifications to the androgen receptor. BJU Int 95: 1320–1326 15892825 10.1111/j.1464-410X.2005.05526.x

[bib10] Edwards J, Krishna NS, Grigor KM, Bartlett JM (2003) Androgen receptor gene amplification and protein expression in hormone refractory prostate cancer. Br J Cancer 89: 552–556 12888829 10.1038/sj.bjc.6601127 PMC2394367

[bib11] Festuccia C, Gravina GL, Angelucci A, Millimaggi D, Muzi P, Vicentini C, Bologna M (2005) Additive antitumor effects of the epidermal growth factor receptor tyrosine kinase inhibitor, gefitinib (Iressa), and the nonsteroidal antiandrogen, bicalutamide (Casodex), in prostate cancer cells *in vitro*. Int J Cancer 115: 630–640 15700310 10.1002/ijc.20917

[bib12] Ghosh PM, Malik SN, Bedolla RG, Wang Y, Mikhailova M, Prihoda TJ, Troyer DA, Kreisberg JI (2005) Signal transduction pathways in androgen-dependent and -independent prostate cancer cell proliferation. Endocr Relat Cancer 12: 119–134 15788644 10.1677/erc.1.00835

[bib13] Gregory CW, He B, Johnson RT, Ford OH, Mohler JL, French FS, Wilson EM (2001a) A mechanism for androgen receptor-mediated prostate cancer recurrence after androgen deprivation therapy. Cancer Res 61: 4315–4319 11389051

[bib14] Gregory CW, Johnson Jr RT, Mohler JL, French FS, Wilson EM (2001b) Androgen receptor stabilization in recurrent prostate cancer is associated with hypersensitivity to low androgen. Cancer Res 61: 2892–2898 11306464

[bib15] Habib FK, Lee IR, Stitch SR, Smith PH (1976) Androgen levels in the plasma and prostatic tissues of patients with benign hypertrophy and carcinoma of the prostate. J Endocrinol 71: 99–107 62008 10.1677/joe.0.0710099

[bib16] Handratta VD, Vasaitis TS, Njar VC, Gediya LK, Kataria R, Chopra P, Newman Jr D, Farquhar R, Guo Z, Qiu Y, Brodie AM (2005) Novel C-17-heteroaryl steroidal CYP17 inhibitors/antiandrogens: synthesis, *in vitro* biological activity, pharmacokinetics, and antitumor activity in the LAPC4 human prostate cancer xenograft model. J Med Chem 48: 2972–2984 15828836 10.1021/jm040202w

[bib17] Heracek J, Richard H, Martin H, Starka L, Sachova J, Kuncova J, Eis V, Urban M, Mandys V (2007) Tissue and serum levels of principal androgens in benign prostatic hyperplasia and prostate cancer. Steroids 72: 375–380 17368496 10.1016/j.steroids.2007.01.004

[bib18] Horoszewicz JS, Leong SS, Kawinski E, Karr JP, Rosenthal H, Chu TM, Mirand EA, Murphy GP (1983) LNCaP model of human prostatic carcinoma. Cancer Res 43: 1809–1818 6831420

[bib19] Kaarbø M, Mikkelsen OL, Malerød L, Qu S, Lobert VH, Akgul G, Halvorsen T, Maelandsmo GM, Saatcioglu F (2010) PI3K-AKT-mTOR pathway is dominant over androgen receptor signaling in prostate cancer cells. Cell Oncol 32(1–2): 11–27 20203370 10.3233/CLO-2009-0487 PMC4619056

[bib20] Koivisto P, Kononen J, Palmberg C, Tammela T, Hyytinen E, Isola J, Trapman J, Cleutjens K, Noordzij A, Visakorpi T, Kallioniemi OP (1997) Androgen receptor gene amplification: a possible molecular mechanism for androgen deprivation therapy failure in prostate cancer. Cancer Res 57: 314–319 9000575

[bib21] Lin HK, Hu YC, Lee DK, Chang C (2004) Regulation of androgen receptor signaling by pten (phosphatase and tensin homolog deleted on chromosome 10) tumor suppressor through distinct mechanisms in prostate cancer cells. Mol Endocrinol 18: 2409–2423 15205473 10.1210/me.2004-0117

[bib22] Lin HK, Yeh S, Kang HY, Chang C (2001) Akt suppresses androgen-induced apoptosis by phosphorylating and inhibiting androgen receptor. Proc Natl Acad Sci USA 98: 7200–7205 11404460 10.1073/pnas.121173298 PMC34646

[bib23] Lin J, Adam RM, Santiestevan E, Freeman MR (1999) The phosphatidylinositol 3′-kinase pathway is a dominant growth factor-activated cell survival pathway in LNCAP human prostate carcinoma cells. Cancer Res 59: 2891–2897 10383151

[bib24] Linja MJ, Savinainen KJ, Saramaki OR, Tammela TLJ, Vessella RL, Visakorpi T (2001) Amplification and overexpression of androgen receptor gene in hormone-refractory prostate cancer. Cancer Res 61: 3550–3555 11325816

[bib25] Manin M, Baron S, Goossens K, Beaudoin C, Jean C, Veyssiere G, Verhoeven G, Morel L (2002) Androgen receptor expression is regulated by the phosphoinositide 3-kinease/Akt pathway in normal and tumoral epithelial cells. Biochem J 366: 729–736 11971763 10.1042/BJ20020585 PMC1222812

[bib26] Mohler JL, Gregory CW, Ford III OH, Kim D, Weaver CM, Petrusz P, Wilson EM, French FS (2004) The androgen axis in recurrent prostate cancer. Clin Cancer Res 10: 440–448 14760063 10.1158/1078-0432.ccr-1146-03

[bib27] Mostaghel EA, Page ST, Lin DW, Fazli L, Coleman IM, True LD, Knudsen B, Hess DL, Nelson CC, Matsumoto AM, Bremner WJ, Gleave ME, Nelson PS (2007) Intraprostatic androgens and androgen-regulated gene expression persist after testosterone suppression: therapeutic implications for castration-resistant prostate cancer. Cancer Res 67: 5033–5041 17510436 10.1158/0008-5472.CAN-06-3332

[bib28] Schayowitz A, Sabnis G, Njar VC, Brodie AM (2008) Synergistic effect of a novel antiandrogen, VN/124-1, and signal transduction inhibitors in prostate cancer progression to hormone independence *in vitro*. Mol Cancer Ther 7: 21–32 10.1158/1535-7163.MCT-07-0581 18202015

[bib29] Sun M, Yang L, Feldman RI, Sun XM, Bhalla KN, Jove R, Nicosia SV, Cheng JQ (2003) Activation of phosphatidylinositol 3-kinase/Akt pathway by androgen through interaction of p85{alpha}, androgen receptor, and Src. J Biol Chem 278: 42992–43000 12933816 10.1074/jbc.M306295200

[bib30] Thalmann GN, Anezinis PE, Chang SM, Zhau HE, Kim EE, Hopwood VL, Pathak S, von Eschenbach AC, Chung LW (1994) Androgen-independent cancer progression and bone metastasis in the LNCaP model of human prostate cancer. Cancer Res 54: 2577–2581 8168083

[bib31] Thalmann GN, Sikes RA, Wu TT, Degeorges A, Chang SM, Ozen M, Pathak S, Chung LW (2000) LNCaP progression model of human prostate cancer: androgen-independence and osseous metastasis. Prostate 44: 91–103 10881018 10.1002/1097-0045(20000701)44:2<91::aid-pros1>3.0.co;2-l

[bib32] Titus MA, Schell MJ, Lih FB, Tomer KB, Mohler JL (2005) Testosterone and dihydrotestosterone tissue levels in recurrent prostate cancer. Clin Cancer Res 11: 4653–4657 16000557 10.1158/1078-0432.CCR-05-0525

[bib33] Vasaitis T, Belosay A, Schayowitz A, Khandelwal A, Chopra P, Gediya LK, Guo Z, Fang HB, Njar VC, Brodie AM (2008) Androgen recptor inactivation contributes to antitumor efficacy of 17*α*-hydroxylase/17,20-lyase inhibitor 3*β*-hydroxy-17-(1*H*-benzimidazole-1-yl)androsta-5,16-diene in prostate cancer. Mol Cancer Ther 7: 2348–2357 18723482 10.1158/1535-7163.MCT-08-0230 PMC2643345

[bib34] Vignot S, Faivre S, Aguirre D, Raymond E (2005) mTOR-targeted therapy of cancer with rapamycin derivatives. Ann Oncol 16: 525–537 15728109 10.1093/annonc/mdi113

[bib35] Visakorpi T, Hyytinen E, Koivisto P, Tanner M, Keinänen R, Palmberg C, Palotie A, Tammela T, Isola J, Kallioniemi OP (1995) *In vivo* amplification of the androgen receptor gene and progression of human prostate cancer. Nat Genet 9: 401–406 7795646 10.1038/ng0495-401

[bib36] Yang L, Xie S, Jamaluddin MS, Altuwaijri S, Ni J, Kim E, Chen YT, Hu YC, Wang L, Chuang KH, Wu CT, Chang C (2005) Induction of androgen receptor expression by phosphatidylinositol 3-kinase/Akt downstream substrate, FOXO3a, and their roles in apoptosis of LNCaP prostate cancer cells. J Biol Chem 280: 33558–33565 16061480 10.1074/jbc.M504461200

